# Catatonic Dilemma in a 33-Year-Old Woman: A Discussion

**DOI:** 10.1155/2013/542303

**Published:** 2013-12-12

**Authors:** Alexander Koch, Karin Reich, Jan Wielopolski, Marion Clepce, Marie Fischer, Johannes Kornhuber, Norbert Thuerauf

**Affiliations:** Department of Psychiatry and Psychotherapy, Friedrich-Alexander-University of Erlangen-Nuremberg, Schwabachanlage 6, 91054 Erlangen, Germany

## Abstract

*Case.* We report a case of catatonia with elevated CK, elevated temperature, and hypoferritinemia after abrupt discontinuation of clozapine in a patient with known proneness to catatonic symptoms. Reinstatement of clozapine therapy was contraindicated due to leukopenia. Neuroleptic malign syndrome could not be ruled out by the administration of quetiapine; this prevented the quick use of other potent D2 antagonists. Some improvement was achieved through supportive therapy, high dose of lorazepam, and a series of 10 ECT sessions. Returning to baseline condition was achieved by a very careful increase of olanzapine. *Discussion.* Catatonic symptoms in schizophrenia as well as in NMS might be caused by a lack of striatal dopamine (CS) or dopamine D2 antagonism (NMS). CS might be a “special” kind of schizophrenia featuring both hypo- and hyperactivity of dopaminergic transmission. ECT has been described as a “psychic rectifier” or a “reset for the system.” The desirable effect of ECT in cases of CS might be dopaminergic stimulation in the striatum and decrease of both the dopaminergic activity in the limbic system and the serotonergic activity on 5-HT2 receptors. The desirable effect of ECT in NMS would be explained by activation of dopaminergic transmission and/or liberation of dopaminergic receptors from the causative neuroleptics.

## 1. Introduction

Catatonia is a diagnostic as well as a therapeutic challenge. Clinical syndromes encompass catatonia as a subtype of schizophrenia, catatonia as the prominent feature in malign neuroleptic syndrome, catatonia in malign hyperthermia, akinetic crisis in Parkinson's disease, and motoric symptoms in serotonergic syndrome [1]. In addition, differential diagnoses such as sporadic or iatrogenic parkinsonism, infectious encephalitis (herpes, borrelia, treponema,…), encephalitis associated with NMDA-receptor autoantibodies, limbic encephalitis associated with paraneoplastic antibodies (e.g., Hu, Ri), B12-deficiency with or without anemia/funicular myelosis [2], folate deficiency, multiple sclerosis, and others have to be considered. Common diagnostic criteria in catatonic syndromes are rigor, catalepsy, elevated levels of CK, hyperthermia, dehydration, and leukocytosis. Hypoferritinemia as a diagnostic marker as well as a possible etiologic factor has been proposed but it is not universally accepted.

The most common and urgent diagnostic challenge is the differentiation between catatonic schizophrenia (CS) with febrile/lethal catatonia and neuroleptic malign syndrome (NMS), the so-called catatonic dilemma [3]. Symptoms pointing to NMS are no ability but the will to communicate, shorter prodromal symptoms, more emphasized early extrapyramidal side effects, more severe rigor, less agitation, or aggressive behavior.

Although clinical features of both conditions can be extremely similar, their response to medication is not; while catatonia as a symptom of schizophrenia seems to react favorably to (atypical) neuroleptics, especially clozapine, NMS in many cases has an adverse reaction to them and might be indistinguishable from akinetic crisis in parkinsonism [4]. The clinical role of the ECT for the treatment of NMS has been carefully reviewed [5].

An overly simplified model of schizophrenia states the occurrence of “too much dopamine” at some point and therefore would infer “too much dopamine” in catatonia. An overly simplified model of neuroleptics regards only their dopamine-antagonistic properties and therefore would infer “too little dopamine” in NMS. Current medical hypotheses offer a synthesis for those two seemingly completely contrary etiologies.

Both catatonic symptoms in schizophrenia as well as in NMS might be caused by a lack of striatal dopamine (CS) or dopamine D2 antagonism (NMS), that is, “too little dopamine.” In CS there might still be “too much dopamine,” for example, in the limbic system, which explains the tolerance and helpfulness of only atypical neuroleptics in CS, which are known to selectively spare striatal functions and therefore have fewer motoric-adverse effects. In addition, catatonic as well as psychotic symptoms are worsened by activation of 5-HT2-receptors, for which most atypical neuroleptics are antagonists [6]. Dopamine antagonism outside the striatum and selective serotonin antagonism seem to be the major pharmacodynamic correlation for “atypical” properties and the reason for their potential usefulness in CS. Following this line of reasoning, CS might be a “special” kind of schizophrenia in that it features both hypo- and hyperactivity of dopaminergic transmission, for example, in the striatum and in the limbic system. It also sheds further light on the “atypical” properties of neuroleptics and the pathophysiology of schizophrenia itself, especially the involvement of the serotonergic system.

The very same line of reasoning explains the indication and the response to ECT in both CS and NMS, making it a valuable tool in a situation of “catatonic dilemma.” ECT as a very unspecific means of treatment that has been described as a “psychic rectifier,” a “reset for the system,” or a “cleaning of the receptors.” These descriptions refer to the involvement of all major neuronal populations in a short, excessive stimulation, leading to mitigation of differences in activity. Hypoactive neurons might be activated, hyperactive ones exhausted, and receptor sensitization or desensitization to previous medication be reversed. The desirable effect of ECT in cases of CS might therefore be in the mitigation of differences in activity, that is, dopaminergic stimulation in the striatum and decrease of both dopaminergic activity in the limbic system and serotonergic activity on 5-HT2-receptors. The desirable effect of ECT in NMS would be explained by activation of dopaminergic transmission and/or liberation of dopaminergic receptors from the causative neuroleptics. An effect on other causative-neurotransmitter systems is also probable.

Regarding additional mechanisms of CS and NMS, the involvement of glutamate, GABA, and acetylcholine in the pathogenesis of catatonic symptoms can be surmised, as their respective antagonists have been shown to have a therapeutic effect. Remaining glutamatergic activity in the basal ganglia and in the hypothalamus during dopamine depletion is thought to be responsible for rigor and hyperthermia; NMDA antagonists exert muscle-relaxant and temperature-decreasing effects. In contrast, anticholinergics have ambivalent effects, since they might decrease muscle tone but also prevent heat dissipation and induce delirium [4].

All specific medications for catatonia in schizophrenia and NMS can be justified with only anecdotal evidence; a review of case reports stated that there is still no evidence-based medication [4]. Exceptions might be conditions of clear etiology such as akinetic crisis following withdrawal of dopaminergic medication or anesthetic-induced malign hyperthermia.

In conclusion, trigger factors of catatonia of many etiologies have been described. Single causes such as the use of potent neuroleptics are unlikely to produce clinical illness; relatively high receptor occupation even in long-lasting therapies without incidents as well as catatonia without concurrent use of neuroleptics suggests other prerogative factors, such as increased heat load, impaired heat dissipation, dehydration, old age, or preexisting brain damage [7].

In psychiatric clinics the most relevant distinction has to be made between febrile/lethal catatonia and neuroleptic malign syndrome. Diagnosis is still mostly made within the simple hypothetic framework of “too much dopamine” and “not enough dopamine,” which might be enough for emergency situations. Therefore, recent withdrawal of neuroleptic medication would be consistent with “schizophrenic” catatonia, while recent increases in dosage or change of neuroleptic medication would warrant the treatment of NMS.

## 2. Case

A 33-year-old woman of Greek origin was admitted to the hospital with mutism, immobility, rigor, waxy flexibility of the limbs, and withdrawal. She had been suffering from paranoid schizophrenia for 15 years, with catatonic symptoms on several occasions. Her last stable medication consisted of 250 mg clozapine per day for six months.

She had been treated in a peripheral hospital for four weeks prior to admission. Her initial symptoms had consisted of delusional perception and ideas, social inadequacy, medical incompliance, disorganization, and self-endangerment when driving a car; yet she had claimed to be “perfectly well.” Blood tests revealed significant leukopenia (1300/*μ*L), and the clozapine medication was stopped abruptly. During the course of two weeks she developed catatonic symptoms, as described on admission. Quetiapine as a substitute for clozapine did not alleviate the symptoms, only lorazepam i.v. showed some benefit at up to 8 mg/d.

Computed tomography revealed no striking organic correlation; no focal lesions of any kind and no irregularities of the ventricular system could be found. Only frontal hyperostosis and marked frontotemporal cortical sulci were present, consistent with chronic schizophrenia.

CSF testing was done in order to rule out an infectious or autoimmune encephalitic etiology. Levels of IgA, IgM, and IgG in CSF and serum as well as their respective ratios were normal. Borrelia-specific antibodies were negative. Proteins, cells, glucose, and lactate were normal.

Blood testing performed in the peripheral clinic initially showed leukopenia, which recuperated quickly after clozapine was stopped. Slight anemia suggested long-term effects during the months before. Ferritin levels were very low (10 ng/mL), indicating concurrent Hypoferritinemia. CRP was normal. About ten days after the clozapine withdrawal, creatine kinase in serum markedly increased, showing a peak of about 2000 U/L after another five days. Body temperature increased only slightly (max. 38.3°C). EEG and ECG yielded no pathological results.

A definite diagnosis could not be made on admission to our hospital; catatonic development following the discontinuation of clozapine suggested schizophrenic catatonia, yet neuroleptic malignant syndrome could not be ruled out, since other neuroleptics had been used thereafter. This catatonic dilemma justified the electroconvulsive therapy combined with only lorazepam.

The patient initially received ECTs on two consecutive days, followed by a full series of ten ECTs at a frequency of two per week. After the first 2–4 days in the hospital, some improvement could be seen, such as episodic speech, beginning of motoric activity, and even walking. However, symptoms remained extremely volatile, ranging from short adequate statements “I am thirsty” and intentional movement to complete withdrawal with rigor and waxy flexibility. Oral nutrition was insufficient. Liquids had to be substituted intravenously from the very beginning, and parenteral feeding started after the first week.

After about four weeks and 10 ECT sessions, the patient was able to drink and eat sufficiently, yet catatonic symptoms were still present, albeit intermittently. She showed no motivation nor initiative to perform everyday tasks but could be guided reasonably well. In addition, she had developed psychotic symptoms with optical hallucinations and expressions of fear. Since no more vital threat seemed imminent, a course of olanzapine was begun, starting with 2.5 mg/d and very slowly increasing to 15 mg/d. Concurrently, lorazepam was slowly tapered and citalopram was administered at up to 20 mg/d. Following this regimen the patient's condition markedly improved; the catatonic symptoms were alleviated and stabilized at a tolerable level. Psychotic perception virtually ceased and activity increased, though guidance remained necessary. Questioning of the patient's father and medical reference persons suggested no better results at baseline. After about nine weeks of treatment, the patient could be transferred to an open asylum.

## 3. Discussion

We report a case of catatonia with elevated CK, elevated temperature and Hypoferritinemia after abrupt discontinuation of clozapine in a patient with known proneness to catatonic symptoms. Reinstatement of clozapine therapy was contraindicated due to leukopenia. Neuroleptic malign syndrome could not be ruled out due to the administration of quetiapine in a peripheral clinic; this prevented the quick use of more potent D2 antagonists, that is, virtually all neuroleptics. Some improvement was achieved by supportive therapy—above all permissive i.v. fluid substitution—, high-dose lorazepam, and a series of 10 ECT sessions. Returning to baseline condition was achieved by very careful increase of olanzapine.

The theoretical background underlying our therapeutic efforts is the complex interplay of several neurotransmitter systems at different anatomic locations and via different types of receptors; this offers a synthesis for seemingly contradictory observations and might further elucidate the pathophysiology of (catatonic) schizophrenia and NMS.
